# Supplementary material for: Content-rich biological network constructed by mining PubMed abstracts
Source: BMC Bioinformatics. 2004 Oct 8;5:147. doi: 10.1186/1471-2105-5-147 (PMC528731; doi:10.1186/1471-2105-5-147)
Supplement: Additional File 2 — The original results of the above study (non-essential files are deleted to keep the file size under the limit set by BMC bioinformatics). [file 1471-2105-5-147-S2.bz2 › chilibotAdditionalFile2/dip05/276ID1827203E1096/html/ATF4_JUN.html]

 


 **ATF4** and **JUN** 
  
Found 16 abstracts in PubMed, retrieved 05.  
 

 What does Google say? 
 PDF only 
| .edu only 

---

**Interactive relationship** (e.g. stimulation, inhibition, etc)

**Neutral relationship**- In contrast, a dominant negative CREB2  [ **ATF4** ]  mutant did not impair ATF2 mediated transcriptional activation, suggesting that CREB2  [ **ATF4** ]  exhibits a different dimerization specificity than ATF2 or c  **Jun** .  Ref: 12751796 Biol Chem, 2003

**Non-interactive relationship** (e.g. studied together, co-existance, homology, etc.)

- Binding of CREB 2  [ **ATF4** ] , ATF 2, USF 2, and c  **Jun**  transactivators to a wild type and CRE mutated oligonucleotide was analyzed by a novel DNA binding assay.  Ref: 12057991 Circulation, 2002
- Antibody supershift EMSA and Western blot analysis confirmed that increased binding of these factors correlated with LPS induced increases in nuclear concentrations of AP 1  [ **JUN** ]  c  **Jun** , phosphorylated c  **Jun** ,  **Jun**  D, and  **Jun**  B, C EBPbeta, NF kappaB p50, p65, and c Rel, CREB CREB 1, CREB 2  [ **ATF4** ] , and ATF 2, and AP 2alpha proteins.  Ref: 12662898 Toxicol Appl Pharmacol, 2003
